# Supplementary material for: Mycobacterium tuberculosis progresses through two phases of latent infection in humans
Source: Nat Commun. 2020 Sep 25;11:4870. doi: 10.1038/s41467-020-18699-9 (PMC7519141; doi:10.1038/s41467-020-18699-9)
Supplement: Supplementary file 1 — Supplementary Information [file 41467_2020_18699_MOESM1_ESM.pdf]

***Mycobacterium tuberculosis* progresses through two phases of latent infection in humans.**

Supplementary Information

Roberto Colangeli[a*], Aditi Gupta[a*], Solange Alves Vinhas[b*], Uma Deepthi Chippada Venkata[a], Soyeon Kim[c], Courtney Grady[a], Edward C. Jones-López[d], Patricia Soteropoulos[e], Moisés Palaci[b], Patrícia Marques-Rodrigues[b], Padmini Salgame[a], Jerrold J. Ellner[a], Reynaldo Dietze[b], and David Alland[#a]

[a] Department of Medicine, Rutgers-New Jersey Medical School, Newark, NJ, USA.

[c] Núcleo de Doenças Infecciosas, Universidade Federal do Espírito Santo (UFES), Vitória, Brazil.

[c] Frontier Science Foundation. 1371 Beacon Street, Suite #203. Brookline, MA 02446, USA.

[d] Division of Infectious Diseases, Department of Medicine. Keck School of Medicine of USC. University of Southern California Los Angeles, CA, USA.

[e] The Genomics Center, Rutgers-New Jersey Medical School, Newark, NJ, USA.

*contributed equally

[#]Address request for reprints to David Alland: allandda@njms.rutgers.edu.

SUPPLEMENTARY METHODS

Modeling new SNP incidence during progression of LTBI

While our data shows that new SNPs are unlikely to arise in a continuing latent infection, suggesting that the bacteria enters a quiescent state, the time to this bacterial adaptation of entering a quiescent state is not clear. To simplify the analysis to answer this question, we simulated SNPs arising every 6 months during LTBI under the following two scenarios: 1) all new SNPs arise during active replication immediately post-infection, a period we call "early latency" in this analysis, and 2) all new SNPs arise during the active replication immediately prior to "reactivation" that leads to an active infection at the end of LTBI. For the "early latency" scenario, we assumed that new SNP incidence in each subsequent six-month time interval during LTBI to be:

i) Constant, i.e. similar number of new SNPs arise in each six-month time interval during LTBI. This is modeled as $n = n_{t0} \times \exp(0)$, where n is the number of new SNPs accrued in a six-month time interval *t*, n_{t0} is the average number of SNPs accrued by the HHC in the four IC-HHC pairs where the HHC was diagnosed within six months of IC (14.5 SNPs, as per SNPTB pipeline), and *t* is the time interval between IC and HHC diagnosis. Since the SNP incidence is modeled for each six-month time interval, there are a total of 11 time intervals in our data with *t* ranging from 0 to 10.

ii) Linear decline in new SNP incidence in each subsequent six-month interval during LTBI, modeled as $= (-1 \times \frac{n_{t0}}{11} \times t) + n_{t0}$.

iii) Exponential decline in new SNP incidence in each subsequent six-month interval during LTBI, modeled as $n = n_{t0} \times \exp(-1 \times t)$.

iv) Rapid exponential decline in new SNP incidence in each subsequent six month interval during LTBI, modeled as $n = n_{t0} \times \exp(-10 \times t)$.

These four scenarios of changes in new SNP incidence over time during LTBI is shown in Supplementary Figure S10-a.

For the "reactivation" scenario, we hypothesized new SNP incidence in each subsequent six-month time interval during LTBI to be (see Figure S10-b):

i) Constant, i.e. similar number of new SNPs arise in each six-month interval of LTBI as in the last six-month interval immediately prior to reactivation, modeled as $n = n_{t0} \times \exp(0)$.

ii) Linear increase, i.e. number of new SNPs in each six-month interval during LTBI increases linearly till reactivation, modeled as $n = 1.46 \times t$.

iii) Exponential increase in new SNP incidence in each subsequent six-month interval, suggesting that majority of SNPs arise in the ultimate and penultimate six-month intervals prior to reactivation, modeled as $n = 10^{-3} \times \exp(0.96 \times t)$

iv) Rapid exponential increase in new SNP incidence, suggesting that majority of new SNPs arise in the six-month interval immediately prior to reactivation, modeled as $n = 10^{-22} \times \exp(5.33 \times t)$.

Since mutation occurrence is rare and is modeled as a Poisson distribution, we randomly sampled mutation incidence per six month time interval from the Poisson distributions (using the numpy.random.poisson function in the SciPy package) with SNP incidence rates as shown in Figure S10a-b. For example, the "constant" hypothesis used the rate parameter λ=14.5 in each time interval. The Poisson distribution rate parameters for the other hypotheses were obtained from Figure S10a-b.

The total SNP accumulation for each IC-HHC pair during LTBI was simulated as follows:

(1)

$$s_i = \sum_{j=1}^{i} x \sim Poisson(\lambda_j)$$

where, i is the total number of six-month intervals spanning IC and HHC diagnosis (=duration of latency) for this pair, and x is a random sample from a Poisson distribution with rate λ_j.

A thousand simulations were performed for each scenario (constant, linear, exponential and rapid exponential) in each scenario (early latency and reactivation), keeping the latency durations and dataset size identical to the observed data. Each simulated dataset was then compared to the observed data using a two-sample two-dimensional Kolmogorov-Smirnov test, and the median two-sided p-value was recorded for each comparison. The script for performing the two-sample two-dimensional Kolmogorov-Smirnov test (kstest_2s_2d.m) is available at MATLAB Central. The distributions of these p-values for each hypothesis under each scenario is shown in Figure S10c-d. Data simulated under exponential and rapid exponential models was not statistically significantly different from the observed clinical data, as indicated by the high p-values from the KS test. This suggests that majority of new SNPs during LTBI likely arise during the first six-months post infection, or last six-months prior to reactivation, or both. This analysis further suggests that transition from active replication state to a quiescent state is a rapid one.

Supplementary Figures:

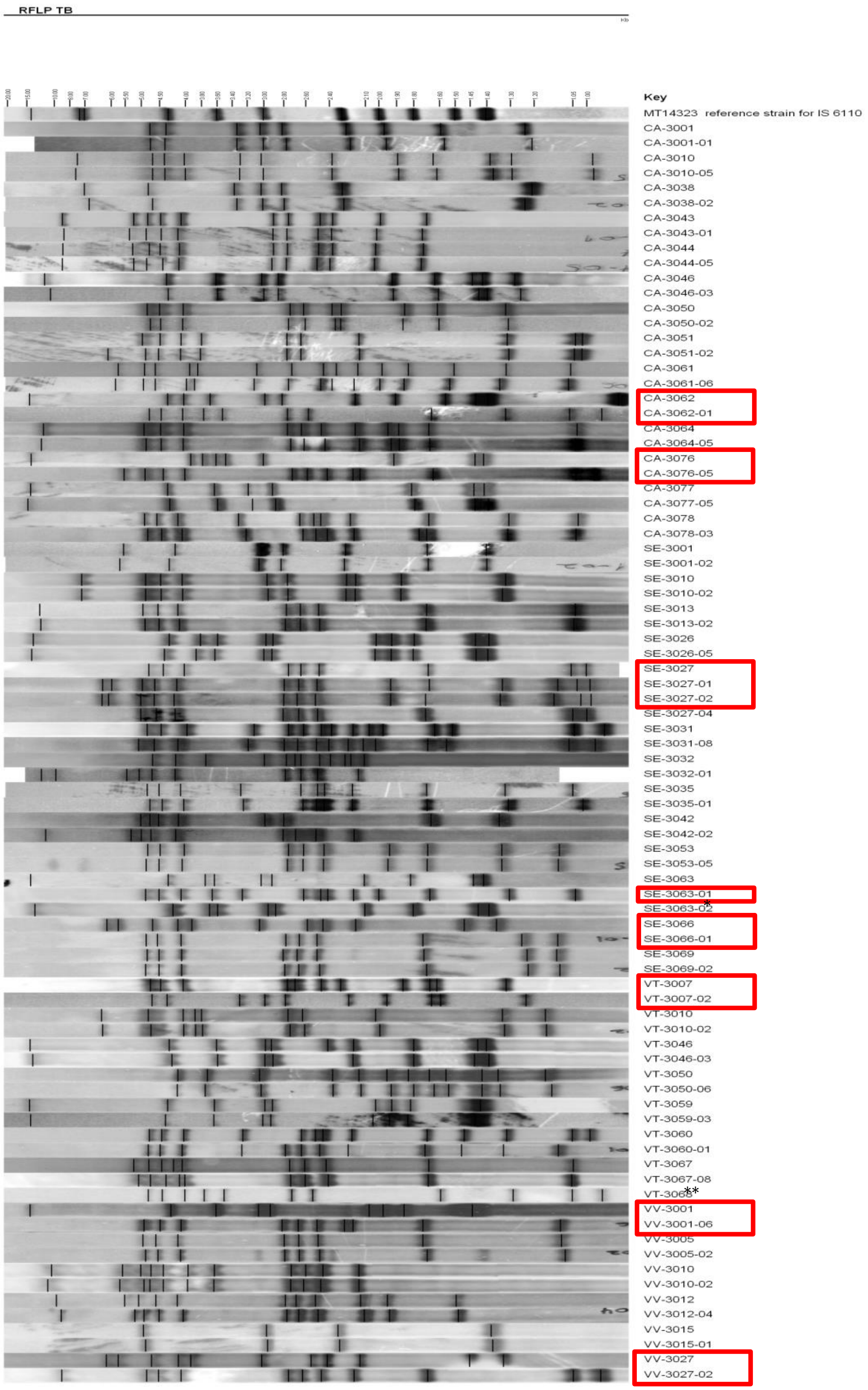

Supplementary Figure 1. Restriction fragment polymorphism (RFLP) analysis. RFLP analysis of a subset of IC and HHC cases. Case numbers in red boxes were excluded from analysis in the study due to mismatched RFLP patterns of the index and HHC (see methods). * Indicates strains in TB pair for patient SE3063. ** Indicates the index strain VT3068. The HHC strain VT3068-2 is not shown. Strain MT14323 is shown in the top lane. This is a standard strain used to provide consistency from gel to gel and it serves as a molecular weight marker for the analysis.

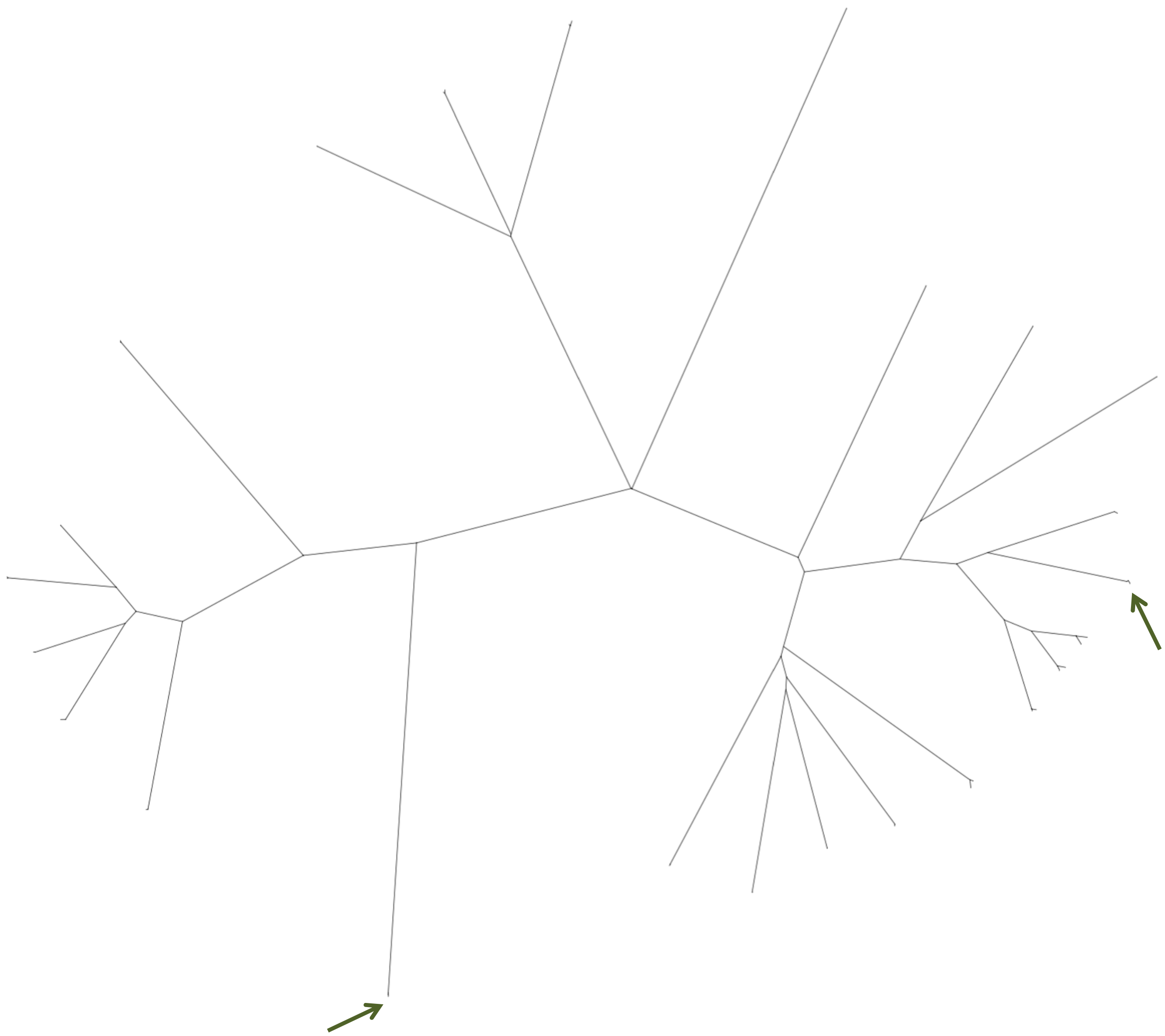

Supplementary Figure 2. Phylogenetically unrelated *M. tuberculosis* isolates. The phylogenetic tree in the Figure 2 of manuscript is shown along with the TB pair whose RFLP patterns matched but was excluded because the IC and HHC isolates did not share a most recent common ancestor (the IC and HHC isolates in this pair are shown by arrows, SNP difference: 748). The phylogenetic tree was drawn using the drawtree program of the PHYLIP package[1]. Source data are provided as a Source Data file.

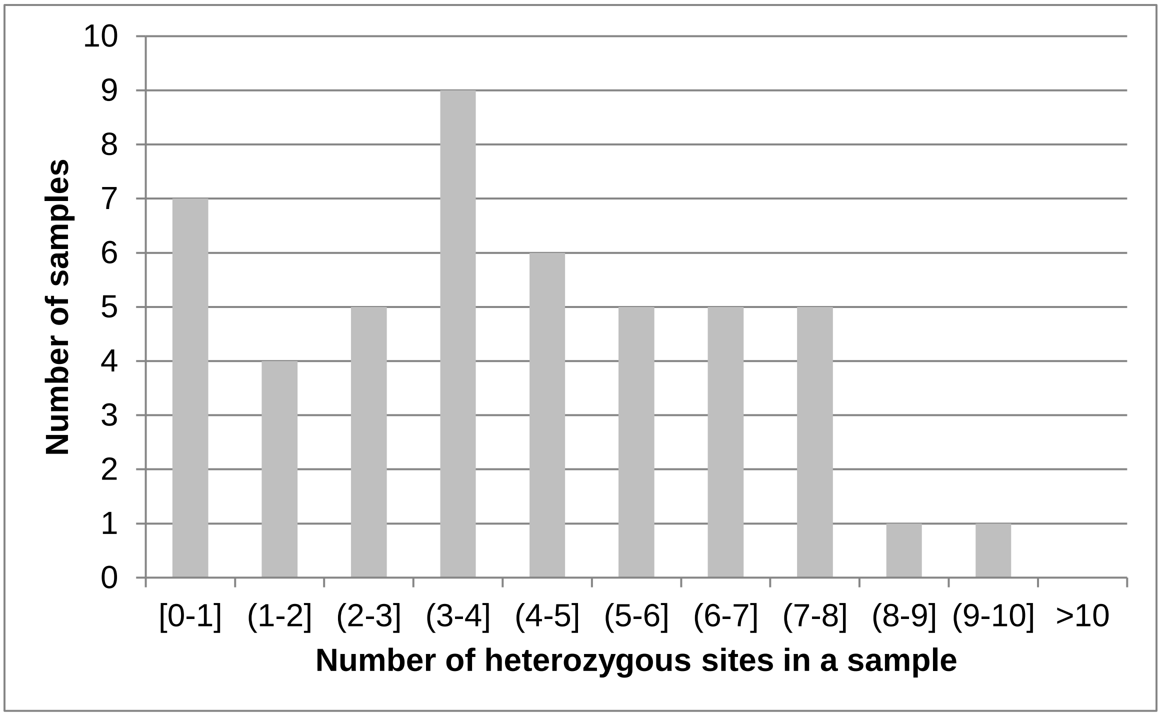

Supplementary Figure 3. Histogram of heterozygous sites in the 24 TB pairs (48 samples). In each sample, the genomic sites with coverage >20 were probed for presence of multiple SNPs such that each SNP is supported by 5% of reads. None of the samples had >10 heterozygous sites, a criteria for potential mixed infection (Sobkowiak *et al.*, BMC Genomics, 2018). Source data are provided as a Source Data file.

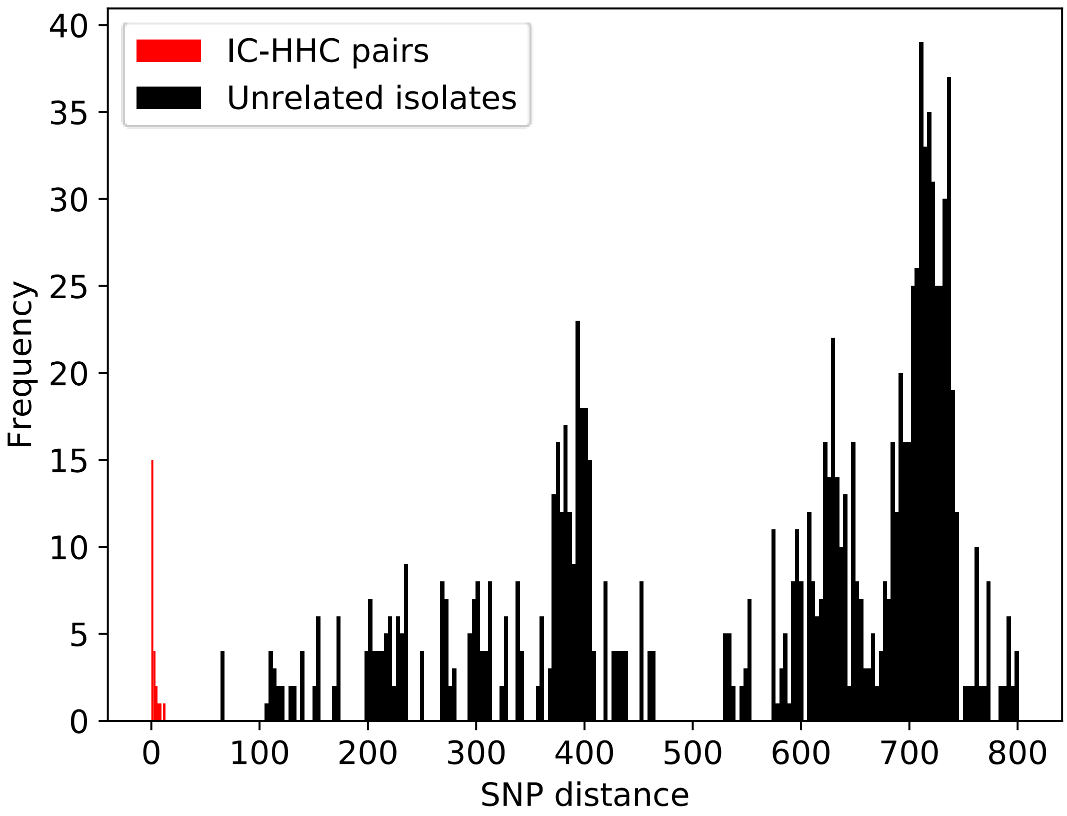

Supplementary Figure 4. SNP differences between IC-HHC pairs and unpaired isolates. The distribution of SNP distances (number of SNPs that are different between two isolates) is shown for the 24 IC-HHC pairs (red, n=24) and 1104 pairings of unrelated index and HHC samples (black, n=1104). The average SNP distance between IC-HHC pairs is 2.25 SNPs and that between unrelated samples is 559.6 SNPs. Source data are provided as a Source Data file.

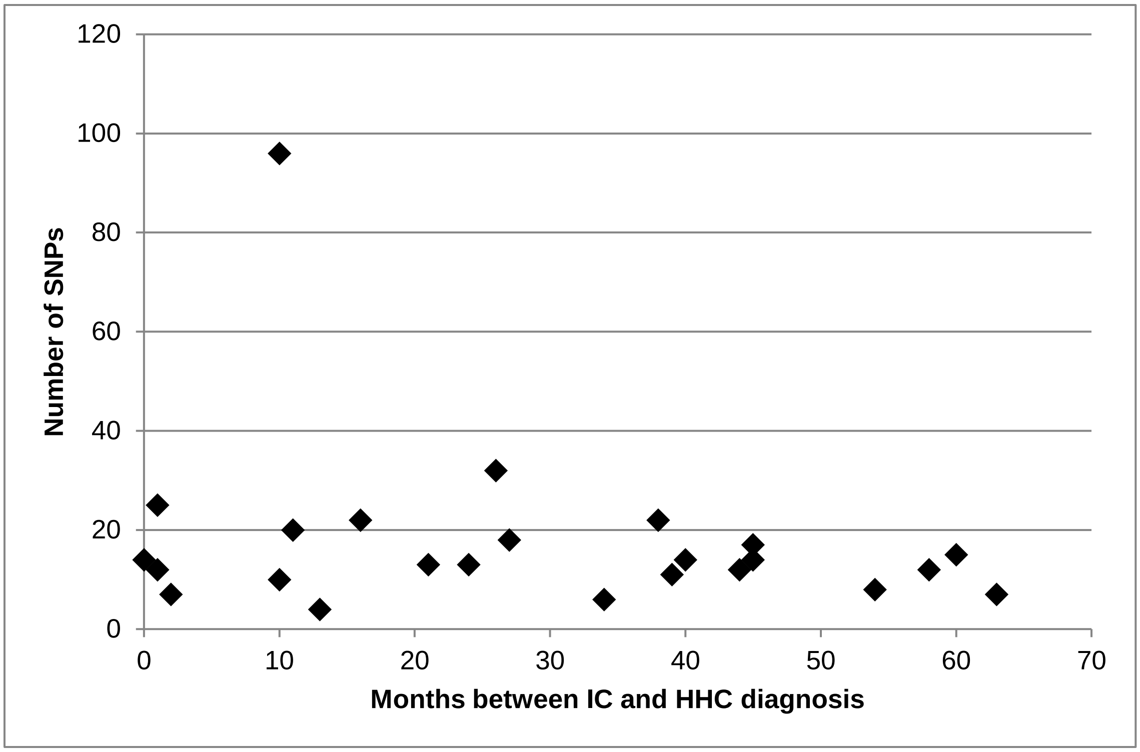

Supplementary Figure 5. SNP difference between IC and HHC using alternate SNP calling pipeline, SNPTB. The scatter plot shows the number of SNPs that are different in the IC and the HHC *M. tuberculosis* isolates when SNPs were called using the SNPTB program for SNP detection (data is shown for n=24 TB pairs). While this pipeline identified more SNPs than the MTBseq pipeline due to less stringent filtering of SNPs (see section "SNP identification" in Methods), the SNP difference does not increase as duration of latency increases, in agreement with the MTBseq analysis. Source data are provided as a Source Data file.

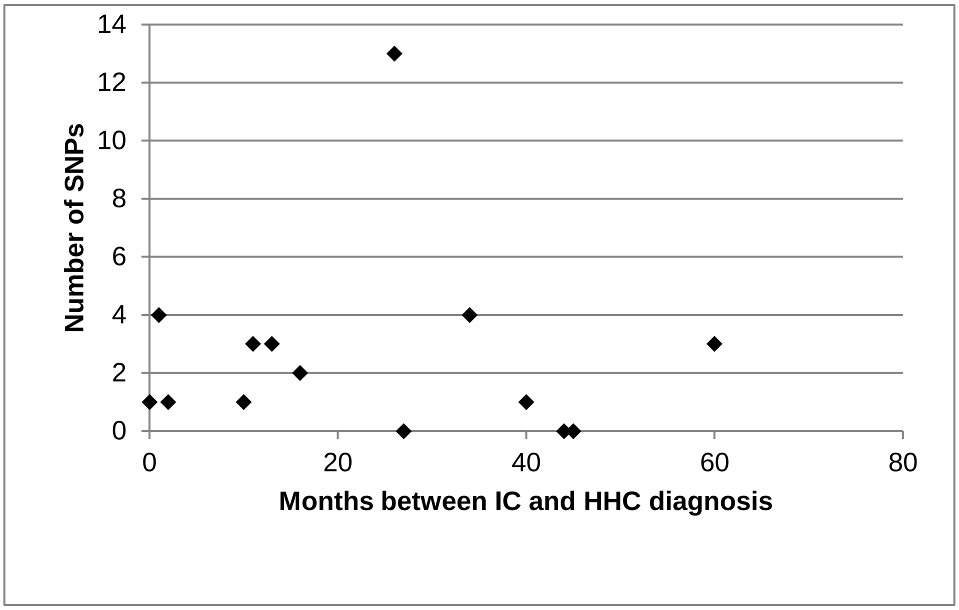

Supplementary Figure 6. SNP accumulation during latency for the 14 TB pairs that had unique RFLP pattern. The scatter plot shows the number of SNPs that differed between the index and the HHC isolates (y-axis) as a function of the duration of *M. tuberculosis* latency (x-axis). Source data are provided as a Source Data file.

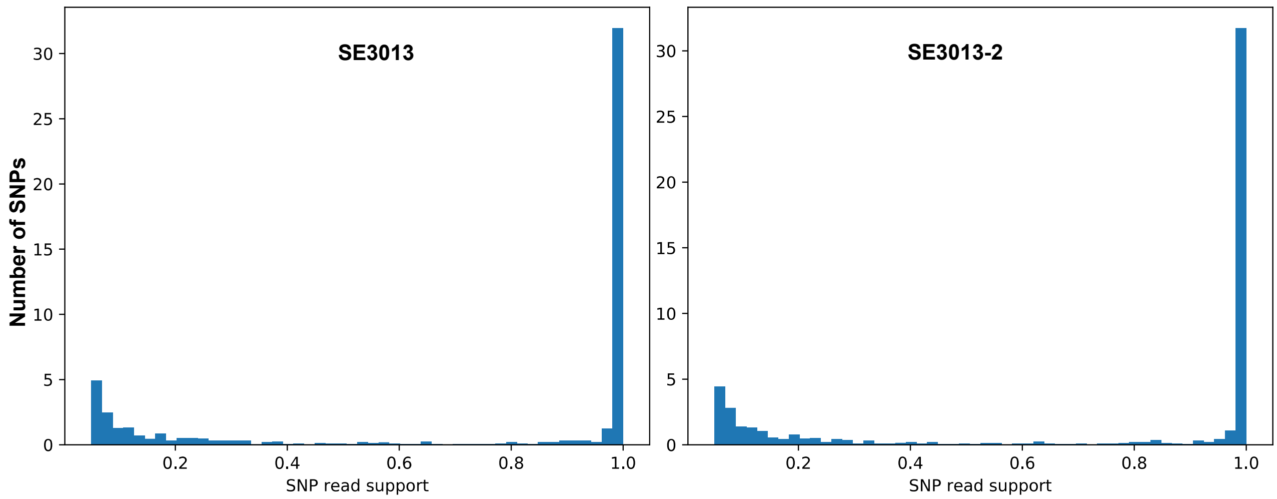

Supplementary Figure 7. Histograms of read support for each SNP in the TB pair SE3013 (IC, left panel) and SE3013-2 (HHC, right panel) is shown. This TB pair had the longest duration of latency in our study (HHC developed active TB infection 63 months after IC diagnosis). Each TB pair had similar SNP read support histograms. This suggests that the bacteria enters a truly quiescent state during LTBI, with no substantial increase in genomic diversity in reactivated patients. The SNP read support of all the samples in this study are similar to the ones shown here. Source data are provided as a Source Data file.

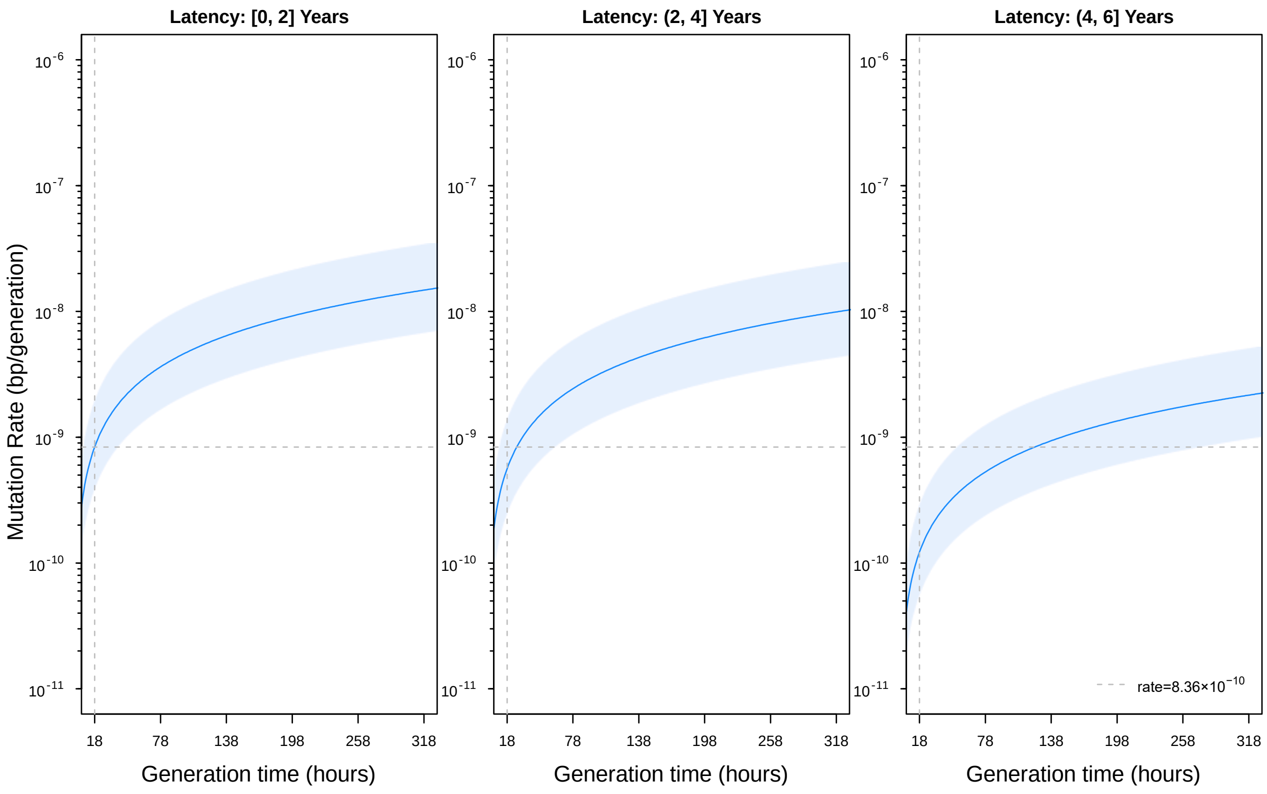

Supplementary Figure 8. Changes in mutation rate during latency with varying generation time. Mutation rate (mutations per (bp×generation)) is shown for generation times ranging from 18 hours to 320 hours for IC-HHC pairs grouped by the number of years between IC diagnosis and reactivation of tuberculosis in the HHC. The latency periods are binned as [0-2], (2-4], and (4-6] years with n=11, 9, and 4 IC-HHC pairs reactivating in the three periods, respectively. For IC-HHC pairs in each of the three latency periods, we fit a Poisson model using an intercept only model using bp×generation as an offset. To test for any difference comparing [0, 2] years, (2, 4] years, and (4, 6] years (a 2 degree of freedom test), we used robust variances to obtain a two-sided chi-square test. The dark blue line shows the average mutation rate per generation time and light blue regions show 95% confidence intervals. The leftmost panel shows the relationship between mutation rate and generation time during early latency (reactivation in ≤2 years of IC diagnosis), with years in latency increasing from left to right. The gray dashed vertical line is at 18 hours and the horizontal line indicates the mutation rate of 8.36×10^{-10} mutations per (bp×generation) as seen in early latency (years 0-2, inclusive) with generation times held constant at 18 hours. The x-axis values where the gray-dashed line intersects the blue line shows the generation times at which that mutation rate is observed as duration of latency increases. The mutation rates decreased from 8.36×10^{-10} [95% confidence interval (CI): 3.71×10^{-10}, 1.88×10^{-9}] mutations per (bp×generation) in ≤2 years of latency, to 5.61×10^{-10} [95% CI: 2.37×10^{-10}, 1.33×10^{-9}] mutations per (bp×generation) in >2 to 4 year-long LTBI, to 1.22×10^{-10} [95% CI: 5.34×10^{-11}, 2.80×10^{-10}] mutations per (bp×generation) in >4 to 6 year-long LTBI (p=0.003). Source data are provided as a Source Data file.

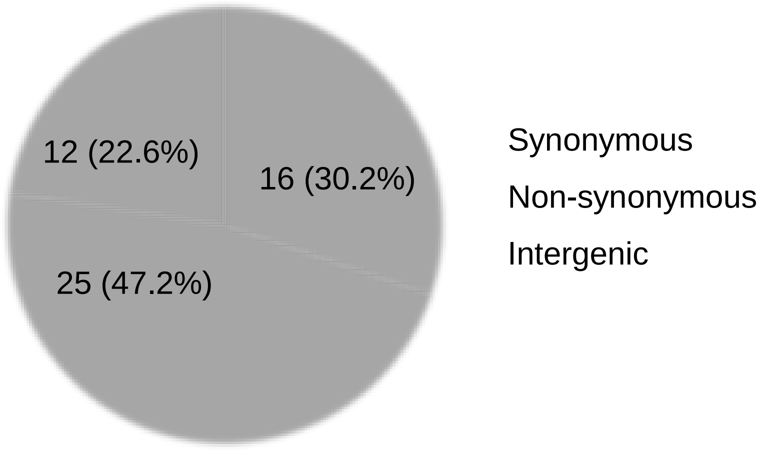

Supplementary Figure 9. Distribution of mutation effects. Of the 54 SNPs that differed between bacterial isolates from all IC-HHC pairs, the majority (47%) were non-synonymous. Source data are provided as a Source Data file.

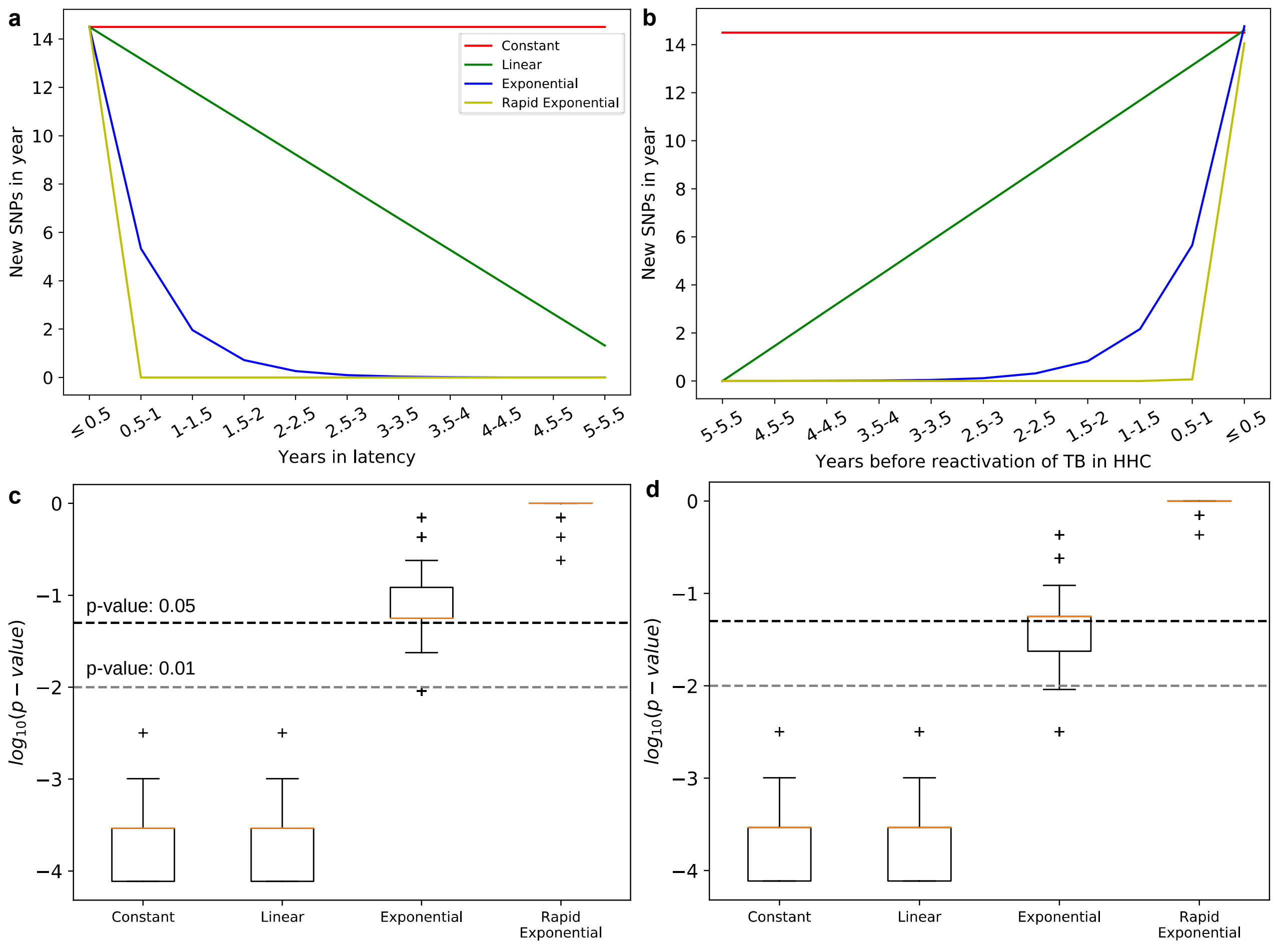

Supplementary Figure 10. Modeling changes in new SNP incidence during LTBI. Four hypotheses of new SNP incidence in each subsequent six-month interval during latency are shown for scenario where early latency contributes majority of SNPs (a) and for scenario where most SNPs are acquired during the reactivation of latent bacteria (b). The four hypotheses of changes in new SNP incidence during latency are: i) Constant SNP accumulation throughout latency (red lines), ii) Linear decline in SNP accumulation in early latency model and linear increase in SNP accumulation in the reactivation model (green lines), iii) exponential decline in new SNP incidence in early latency model and exponential increase during reactivation (blue lines), and iv) a rapid exponential decline in new SNP incidence in early latency and rapid exponential increase during reactivation (yellow lines). All hypotheses start with the assumption that, on average, 14.5 SNPs arise in the first six-months of latency, as was the case in the observed data. Thereafter, different hypotheses accrue SNPs in each subsequent time interval according to their respective decay or growth functions shown in panels a-b. The distribution of p-values of comparing each of the 1000 simulated datasets per hypothesis with the observed SNP incidence data is shown as boxplots for early latency model (c) and the reactivation model (d). For panels (c) and (d), n=1000, where each $\log_{10}$(two-sided p-value) was obtained by comparing each simulated set of SNP values to the observed data using the two-sample two-dimensional K-S test. For panels (c) and (d), the lower whisker is at the lowest data point above Q1 – 1.5*(Q3-Q1), and the upper whisker is at the highest data point below Q3 + 1.5*(Q3-Q1), where Q1 and Q3 are the first and third quartiles; the black box shows the middle 50% of the data; the

orange line shows the median value; the outliers are shown as ‘+’ sign. The black dashed line denotes p-value of 0.05 and gray dashed line shows the p-value of 0.01. The exponential model and the rapid exponential model simulate SNP incidence patterns during LTBI that are statistically indistinguishable from the observed data, as evidenced by high p-values when comparing these simulated data to the observed SNP incidence data. Source data are provided as a Source Data file.

Supplementary Tables:

Supplementary Table 1: Whole-genome sequencing of the IC-HHC pairs yielded an average coverage of 498X across all samples. The number of mapped reads per sample ranged from 9.65 million to 20.4 million reads, with an average of 16.65 million reads.

Index case	IC average coverage	HHC	HHC average coverage
VV3015	566.11	VV3015-1	506.65
CA3043	378.26	CA3043-1	546.61
SE3001-2	505.98	SE3001	550.15
SE3063	481.18	SE3063-2	503.74
VT3010	461.7	VT3010-2	454.33
VT3050	455.61	VT3050-6	363.53
VT3059	503.84	VT3059-3	356.78
CA3050	595.44	CA3050-2	529.37
CA3061	520.95	CA3061-6	538.79
CA3064	539.67	CA3064-5	578.47
CA3077	558.77	CA3077-5	530.38
VT3046-3	466.29	VT3046	551.9
CA3078-3	551.07	CA3078	509.81
VT3067	522.02	VT3067-8	470.64
CA3001	495.52	CA3001-1	429.73
CA3010	606.93	CA3010-5	468.83
CA3051	553.1	CA3051-2	448.4
SE3010-2	503.91	SE3010	475.14
SE3035-1	425.73	SE3035	495.53
VV3012-4	283.49	VV3012	420.41
CA3046	521.84	CA3046-3	488.04
CA3038	546.12	CA3038-2	603.69
SE3031-8	509.24	SE3031	516.93
SE3013	551.87	SE3013-2	466.08

Supplementary Table 2. Number of mutations that are different between bacterial isolates from the index (IC) and the household contact cases (HHC) using the MTBseq method for SNP detection. The IC-HHC pairs with unique RFLP patterns are highlighted in bold.

Index case	HHC	Years (months) between diagnosis	# SNPs different	# Syn SNPs	# Non-syn SNPs	# SNPs RNA genes	# SNPs intergenic	Observed mutation rate
**VV3015**	**VV3015-1**	**<1 (11)**	**3**	**2**	**1**	**0**	**0**	**1.57E-09**
CA3043	CA3043-1	<1 (10)	0	0	0	0	0	0
**SE3001-2**	**SE3001**	**<1 (1)**	**4**	**0**	**3**	**0**	**1**	**2.30E-08**
**SE3063**	**SE3063-2**	**<1 (2)**	**1**	**1**	**0**	**0**	**0**	**2.87E-09**
**VT3010**	**VT3010-2**	**<1 (0)**	**1**	**1**	**0**	**0**	**0**	**5.75E-09***
**VT3050**	**VT3050-6**	**<1 (10)**	**1**	**0**	**0**	**0**	**1**	**5.75E-10**
VT3059	VT3059-3	<1 (1)	1	0	1	0	0	5.75E-09
CA3050	CA3050-2	1-2 (21)	0	0	0	0	0	0
CA3061	CA3061-6	1-2 (24)	0	0	0	0	0	0
**CA3064**	**CA3064-5**	**1-2 (16)**	**2**	**0**	**1**	**0**	**1**	**7.18E-10**
**CA3077**	**CA3077-5**	**1-2 (13)**	**3**	**1**	**2**	**0**	**0**	**1.33E-09**
**VT3046-3**	**VT3046**	**2-3 (27)**	**0**	**0**	**0**	**0**	**0**	**0**
**CA3078-3**	**CA3078**	**2-3 (26)**	**13**	**4**	**5**	**1**	**3**	**2.87E-09**
**VT3067**	**VT3067-8**	**2-3 (34)**	**4**	**1**	**0**	**0**	**3**	**6.76E-10**
CA3001	CA3001-1	3-4 (45)	0	0	0	0	0	0
**CA3010**	**CA3010-5**	**3-4 (40)**	**1**	**1**	**0**	**0**	**0**	**1.44E-10**
CA3051	CA3051-2	3-4 (39)	7	2	4	0	1	1.03E-09
**SE3010-2**	**SE3010**	**3-4 (44)**	**0**	**0**	**0**	**0**	**0**	**0**
SE3035-1	SE3035	3-4 (38)	8	3	4	0	1	1.21E-09
**VV3012-4**	**VV3012**	**3-4 (45)**	**0**	**0**	**0**	**0**	**0**	**0**
CA3046	CA3046-3	4-5 (58)	1	0	1	0	0	9.91E-11
**CA3038**	**CA3038-2**	**4-5 (60)**	**3**	**0**	**3**	**0**	**0**	**2.87E-10**
SE3031-8	SE3031	4-5 (54)	0	0	0	0	0	0
SE3013	SE3013-2	5-6 (63)	1	0	0	0	1	9.12E-11
		**Average**	2.25	0.67	1.04	0.04	0.5	
		**Total**	54	16	25	1	12	

*HHC diagnosis conservatively assumed to occur 1 month after that of the Index Case for the calculation of observed rate.

Supplementary Table 3. Number of mutations that are different between bacterial isolates from the index (IC) and the house-hold contact cases (HHC) using the SNPTB method for SNP detection.

Index case	HHC	Years (months) between diagnosis	# SNPs different	# Syn SNPs	# Non-syn SNPs	# SNPs RNA genes	# SNPs intergenic
VV3015	VV3015-1	<1 (11)	20	10	8	0	2
CA3043	CA3043-1	<1 (10)	10	6	2	0	2
SE3001-2	SE3001	<1 (1)	25	7	12	0	6
SE3063	SE3063-2	<1 (2)	7	3	4	0	0
VT3010	VT3010-2	<1 (0)	14	3	6	0	5
VT3050	VT3050-6	<1 (10)	96	28	49	0	19
VT3059	VT3059-3	<1 (1)	12	6	6	0	0
CA3050	CA3050-2	1-2 (21)	13	5	5	0	3
CA3064	CA3064-5	1-2 (24)	13	3	7	0	3
CA3077	CA3077-5	1-2 (16)	22	8	14	0	0
CA3061	CA3061-6	1-2 (13)	4	1	1	0	2
VT3046-3	VT3046	2-3 (27)	18	11	4	0	3
CA3078-3	CA3078	2-3 (26)	32	12	12	0	8
VT3067	VT3067-8	2-3 (34)	6	2	2	0	2
CA3001	CA3001-1	3-4 (45)	14	2	7	0	5
CA3010	CA3010-5	3-4 (40)	14	5	6	0	3
CA3051	CA3051-2	3-4 (39)	11	2	7	0	2
SE3010-2	SE3010	3-4 (44)	12	2	6	0	4
SE3035-1	SE3035	3-4 (38)	22	7	8	0	7
VV3012-4	VV3012	3-4 (45)	17	7	7	0	3
CA3046	CA3046-3	4-5 (58)	12	4	7	0	1
SE3031-8	SE3031	4-5 (60)	15	4	8	0	3
CA3038	CA3038-2	4-5 (54)	8	2	5	0	1
SE3013	SE3013-2	5-6 (63)	7	4	0	0	3
		**Average**	17.67	6	8.04	0	3.625
		**Total**	424	144	193	0	87

Supplementary Table 4. *M. tuberculosis* genes that had non-synonymous SNPs are indicated along with the number of IC-HHC pairs that had such mutations.

Gene	Gene Description (source: NCBI Gene database)	# IC-HHC pairs with mutation in the gene
esxL_Rv1198	ESAT-6 like protein EsxL	1
_Rv1318c	adenylate cyclase	1
_Rv0575c	oxidoreductase	1
_Rv1230c	membrane protein	1
ppm1_Rv2051c	polyprenol-monophosphomannose synthase	1
cyp125_Rv3545c	steroid C26-monooxygenase	1
bioF2_Rv0032	8-amino-7-oxononanoate synthase	1
glpK_Rv3696c	glycerol kinase	1
mbtE_Rv2380c	peptide synthetase	1
ltp1_Rv2790c	lipid-transfer protein	1
_Rv0386	transcriptional regulator	1
ctpG_Rv1992c	cation transporter ATPase G	1
glbO_Rv2470	hemoglobin GlbO	1
_Rv2734	hypothetical protein	1
_Rv3402c	hypothetical protein	1
_Rv0712	hypothetical protein	1
_Rv3242c	hypothetical protein	1
fadD29_Rv2950c	long-chain-fatty-acid--AMP ligase FadD29	1
_Rv2252	diacylglycerol kinase	1
_Rv3401	glycosyl hydrolase	1
_Rv0464c	hypothetical protein	1
fadD14_Rv1058	fatty-acid--CoA ligase FadD14	1
qcrA_Rv2195	ubiquinol-cytochrome C reductase rieske iron-sulfur subunit	1
thiC_Rv0423c	phosphomethylpyrimidine synthase	1
_Rv2015c	hypothetical protein	1

Supplementary Table 5. *M. tuberculosis* genes that had synonymous SNPs are indicated along with the number of IC-HHC pairs that had such mutations.

Gene	Gene Description (source: NCBI Gene database)	# IC-HHC pairs with mutation in the gene
folK_Rv3606c	2-amino-4-hydroxy-6-hydroxymethyldihydropteridinepyrophosphokinase	1
aroA_Rv3227	3-phosphoshikimate 1-carboxyvinyltransferase	1
_Rv2086	hypothetical protein	1
dinP_Rv3056	DNA polymerase IV 2	1
ctpH_Rv0425c	metal cation transporting ATPase H	1
esxK_Rv1197	ESAT-6 like protein EsxK	1
dapB_Rv2773c	4-hydroxy-tetrahydrodipicolinate reductase	1
nadE_Rv2438c	glutamine-dependent NAD(+) synthetase	1
_Rv0817c	hypothetical protein	1
_Rv1958c	hypothetical protein	1
_Rv3910	peptidoglycan biosynthesis protein	1
_Rv0095c	hypothetical protein	1
_Rv2787	hypothetical protein	1
_Rv0083	oxidoreductase	1
_Rv3635	transmembrane protein	1
gdh_Rv2476c	NAD-dependent glutamate dehydrogenase	1

Supplementary References

1. Felsenstein, J. PHYLIP (Phylogeny Inference Package) version 3.7a. (Distributed by the author. Department of Genome Sciences, University of Washington, Seattle, http://evolution.genetics.washington.edu/phylip/doc/main.html, 2009).
