# Supplementary material for: Mycobacterium tuberculosis progresses through two phases of latent infection in humans
Source: Nat Commun. 2020 Sep 25;11:4870. doi: 10.1038/s41467-020-18699-9 (PMC7519141; doi:10.1038/s41467-020-18699-9)
Supplement: Supplementary file 3 — Reporting Summary [file 41467_2020_18699_MOESM3_ESM.pdf]

nature research

Corresponding author(s): David Alland

Last updated by author(s): Jul 16, 2020

Reporting Summary

Nature Research wishes to improve the reproducibility of the work that we publish. This form provides structure for consistency and transparency in reporting. For further information on Nature Research policies, see our Editorial Policies and the Editorial Policy Checklist.

Statistics

For all statistical analyses, confirm that the following items are present in the figure legend, table legend, main text, or Methods section.

n/a	Confirmed	
☐	☒	The exact sample size (*n*) for each experimental group/condition, given as a discrete number and unit of measurement
☐	☒	A statement on whether measurements were taken from distinct samples or whether the same sample was measured repeatedly
☐	☒	The statistical test(s) used AND whether they are one- or two-sided<br>*Only common tests should be described solely by name; describe more complex techniques in the Methods section.*
☐	☒	A description of all covariates tested
☐	☒	A description of any assumptions or corrections, such as tests of normality and adjustment for multiple comparisons
☐	☒	A full description of the statistical parameters including central tendency (e.g. means) or other basic estimates (e.g. regression coefficient) AND variation (e.g. standard deviation) or associated estimates of uncertainty (e.g. confidence intervals)
☐	☒	For null hypothesis testing, the test statistic (e.g. *F*, *t*, *r*) with confidence intervals, effect sizes, degrees of freedom and *P* value noted<br>*Give P values as exact values whenever suitable.*
☒	☐	For Bayesian analysis, information on the choice of priors and Markov chain Monte Carlo settings
☒	☐	For hierarchical and complex designs, identification of the appropriate level for tests and full reporting of outcomes
☒	☐	Estimates of effect sizes (e.g. Cohen's *d*, Pearson's *r*), indicating how they were calculated

Our web collection on statistics for biologists contains articles on many of the points above.

Software and code

Policy information about availability of computer code

Data collection	No software was used for data collection.
Data analysis	The following softwares were used for data analysis: (1) RFLP band patterns were analyzed using BioNumerics software version 7.6; (2) SNP detection was done using published software MTBseq, code available at https://github.com/ngs-fzb/MTBseq_source; (3) Alternate SNP detection was done using published software SNPTB, code is available at https://github.com/aditi9783/SNPTB; (4) Phylogenetic trees were generated using PHYLIP package version 3.696; (5) Lineage detection was done using published software SNP-IT package available at https://github.com/samlipworth/SNP-IT; (6) Custom scripts to analyze data and generate graphs is available at https://github.com/aditi9783/TB_latency_scripts.

For manuscripts utilizing custom algorithms or software that are central to the research but not yet described in published literature, software must be made available to editors and reviewers. We strongly encourage code deposition in a community repository (e.g. GitHub). See the Nature Research guidelines for submitting code & software for further information.

Data

Policy information about availability of data

All manuscripts must include a data availability statement. This statement should provide the following information, where applicable:

- Accession codes, unique identifiers, or web links for publicly available datasets
- A list of figures that have associated raw data
- A description of any restrictions on data availability

Genomic data (raw sequence reads) from clinical isolates is available from NCBI Sequence Read Archive via BioProject accession number PRJNA475130. H37Rv (laboratory strain) genomic data is available from NCBI Sequence Read Archive via BioProject ID accession number PRJNA607763.

April 2020

Field-specific reporting

Please select the one below that is the best fit for your research. If you are not sure, read the appropriate sections before making your selection.

- [x] Life sciences
- [ ] Behavioural & social sciences
- [ ] Ecological, evolutionary & environmental sciences

For a reference copy of the document with all sections, see nature.com/documents/nr-reporting-summary-flat.pdf

Life sciences study design

All studies must disclose on these points even when the disclosure is negative.

Sample size	This study has a sample size of 24 pairs of index TB cases (IC) and their household contacts - HHC (thus, total 48 total clinical samples). The clinical study (reference 22 in the manuscript) had identified 160 households in Vitoria, Brazil that fit the case definition of containing a highly infectious case of acid-fast bacilli (AFB) smear-positive pulmonary TB, had at least 3 HHCs, and did not fulfill any exclusion criteria. Between the beginning of the HHC study in 2008 and the end of the study in 2015, 72 HHCs associated with 62 of these ICs were found to have developed active TB. A search though the local laboratory database located cultures from 43 of the remaining HHCs with TB, each linked to its known IC, resulting in 43 “TB pairs”. The exclusions described below resulted in the 24 IC-HHC pairs used in the study.
Data exclusions	Figure 1 in the manuscript described TB pairs excluded from the study. Starting from 43 IC-HHC pairs, 13 pairs were excluded due to mismatched RFLP patterns, 2 pairs were excluded because the TB strains were circulating commonly in the community, 2 pairs were excluded because their cultures could not be regrown in the laboratory for DNA extraction, 1 pair was excluded because the whole genome sequencing data yielded insufficient data for analysis, and 1 pair was excluded because phylogenetic analysis revealed the pair to be infected by unrelated TB strains.
Replication	Custom scripts were written to generate the figures and perform data analysis, and thus can be replicated. SNP detection was done using two alternate Bioinformatics pipelines that agreed on the outcomes of the data analyses.
Randomization	The patients are described as index cases for TB or their house contacts that developed TB. All TB pairs that satisfied various inclusion criteria were included in the study. Thus, randomization was not needed.
Blinding	*Describe whether the investigators were blinded to group allocation during data collection and/or analysis. If blinding was not possible, describe why OR explain why blinding was not relevant to your study.*

Reporting for specific materials, systems and methods

We require information from authors about some types of materials, experimental systems and methods used in many studies. Here, indicate whether each material, system or method listed is relevant to your study. If you are not sure if a list item applies to your research, read the appropriate section before selecting a response.

Materials & experimental systems

n/a	Involved in the study	
x		Antibodies
x		Eukaryotic cell lines
x		Palaeontology and archaeology
x		Animals and other organisms
	x	Human research participants
	x	Clinical data
x		Dual use research of concern

Methods

n/a	Involved in the study	
x		ChIP-seq
x		Flow cytometry
x		MRI-based neuroimaging

Human research participants

Policy information about studies involving human research participants

Population characteristics	We obtained M. tuberculosis cultures from participants enrolled in a household contact (HHC) study performed at the Núcleo do Doenças Infecciosas (NDI) located in Vitória, the capital city of the State of Espírito Santo, Brazil. The NDI has organized a network of 16 TB clinics in the metropolitan region of Vitória27, which facilitated the identification of index TB cases as well as secondary TB cases in HHCs after initial ascertainment.
Recruitment	All consecutive HIV-uninfected, pulmonary TB patients attending NDI clinics with a first episode of TB and a sputum with ≥2+ acid-fast bacilli (AFB) smear and ≥3 HHCs who did not meet previously described exclusion criteria were eligible for enrollment as index cases. A HHC was defined using culturally-adapted criteria of close contact. All participating HHCs were enrolled within the first two weeks after the index TB patient had presented to the clinic. Co-prevalent active TB disease was excluded in all HHCs eligible for the current study by a detailed history and sputum examination at the time of the original HHC investigation. Chest-X-rays examination was performed in all participants with symptoms suggestive of TB. To identify secondary cases of TB in the HHCs up to 6 years after identification of the index case, HHC study participants were matched with an extensive clinical and microbiological database "TB notes" of TB cases and M. tuberculosis cultures isolated in the greater Vitória region. This search identified 72 cases of TB in the HHCs. Seven of the HHC were found to have had a case of TB that predated the onset of TB in the "index case" that had led to the HHC investigation. In these 7 cases, the first occurring TB case was defined as the index case and the second occurring TB case was re-defined as the secondary TB case.
Ethics oversight	The study was approved by the Comitê de Ética em Pesquisa do Centro de Ciências da Saúde - Universidade Federal do Espírito Santo and the Comissão Nacional de Ética em Pesquisa under protocol number 14151, and the Institutional Review Boards of Boston University Medical Center and Rutgers University –New Jersey Medical School (formerly University of Medicine and Dentistry of New Jersey). We obtained written informed consent and assent in Portuguese in accordance with age-specific ethical guidelines of participating institutions.

Note that full information on the approval of the study protocol must also be provided in the manuscript.

Clinical data

Policy information about clinical studies

All manuscripts should comply with the ICMJE guidelines for publication of clinical research and a completed CONSORT checklist must be included with all submissions.

Clinical trial registration	The study was approved by the Comitê de Ética em Pesquisa do Centro de Ciências da Saúde - Universidade Federal do Espírito Santo and the Comissão Nacional de Ética em Pesquisa under protocol number 14151.
Study protocol	14151
Data collection	The clinical samples were collected in Vitória, Brazil between February 2008 and October 2013.
Outcomes	*Describe how you pre-defined primary and secondary outcome measures and how you assessed these measures.*
